# Impacts of Respiratory Muscle Training on Respiratory Functions, Maximal Exercise Capacity, Functional Performance, and Quality of Life in School-Aged Children with Postoperative Congenital Diaphragmatic Hernia

**DOI:** 10.1155/2020/8829373

**Published:** 2020-09-04

**Authors:** Samah A. Moawd, Alshimaa R. Azab, Zizi M. Ibrahim, Anju Verma, Walid Kamal Abdelbasset

**Affiliations:** ^1^Department of Physical Therapy for Cardiovascular/Respiratory Disorder and Geriatrics, Faculty of Physical Therapy, Cairo University, Giza, Egypt; ^2^Department of Physical Therapy for Pediatrics, Faculty of Physical Therapy, Cairo University, Giza, Egypt; ^3^Department of Health and Rehabilitation Sciences, College of Applied Medical Sciences, Prince Sattam bin Abdulaziz University, Al-Kharj, Saudi Arabia; ^4^Department of Physical Therapy for Surgery, Faculty of Physical Therapy, Cairo University, Giza, Egypt; ^5^Department of Rehabilitation Sciences, College of Health and Rehabilitation Sciences, Princess Nourah bint Abdulrahman University, Riyadh, Saudi Arabia; ^6^Department of Physical Therapy, Kasr Al-Aini Hospital, Cairo University, Giza, Egypt

## Abstract

**Objectives:**

Congenital diaphragmatic hernia (CDH) is a birth defect affecting the respiratory functions, functional performance, and quality of life (QOL) in school-aged children. Rarely have studies been conducted to evaluate the impacts of respiratory muscle training on school-aged children with postoperative CDH. The current study was designed to evaluate the impacts of respiratory muscle training on respiratory function, maximal exercise capacity, functional performance, and QOL in these children.

**Methods:**

This study is a randomized control study. 40 children with CDH (age: 9-11 years) were assigned randomly into two groups. The first group conducted an incentive spirometer exercise combined with inspiratory muscle training (study group, *n* = 20), whereas the second group conducted only incentive spirometer exercise (control group, *n* = 20), thrice weekly for twelve consecutive weeks. Respiratory functions, maximal exercise capacity, functional performance, and pediatric quality of life inventory (PedsQL) were assessed before and after the treatment program*. Results*. Regarding the posttreatment analysis, the study group showed significant improvements in all outcome measures (FVC%, *p* < 0.001; FEV1%, *p* = 0.002; VO_2_max, *p* = 0.008; VE/VCO_2_ slope, *p* = 0.002; 6-MWT, *p* < 0.001; and PedsQL, *p* < 0.001), whereas the control group did not show significant changes (*p* > 0.05).

**Conclusion:**

Respiratory muscle training may improve respiratory functions, maximal exercise capacities, functional performance, and QOL in children with postoperative CDH. Clinical commendations have to be considered to include respiratory muscle training in pulmonary rehabilitation programs in children with a history of CDH.

## 1. Introduction

Congenital diaphragmatic hernia (CDH) is a birth defect characterized by failure of diaphragmatic muscle closure [1]. The diaphragm is a thin layer of muscle which separates the chest from the abdomen [2]. Accordingly, the content of the abdomen moves from the abdomen to the chest in diaphragmatic hernia [3]. CDH accounts for 1 : 2500 to 1 : 3000 live births [4]. Approximately 80-85% of CDH affects the left side of the diaphragm [5]. According to the site of the diaphragmatic hernias, it is classified into three types: the Bochdalek hernia, the Morgagni hernia, and the central hernia [6]. The Bochdalek hernia occurs in the posterior-lateral part of the diaphragm and affects 70% of the cases, the Morgagni hernia occurs in the anterior part of the diaphragm and affects 25–35% of the cases, and the central hernia occurs in the central part of the diaphragm and affects 2-5% of the cases [6].

Around 50-60% of CDH occur without any other congenital anomalies [7], while 40-50% occur with other congenital anomalies such as craniofacial, ocular, cardiovascular, central nervous system, skeletal, and genitourinary anomalies [8]. Although there is no distinct reason for CDH, it may be associated with multiple genetic factors in concert with environmental and nutritional issues [9–11]. CDH and its related malformation threaten life with a mortality rate ranging from 10 to 50% [12]. Surviving children often present with severe complications such as pulmonary disorders, cardiovascular diseases, microstructural changes in lung gastrointestinal disease, gastroesophageal reflux, and recurrent episodes of lower respiratory tract infections [1, 13–15].

In addition to the previous complication, children with CDH suffer from significant reductions in forced expiratory volume in 1 second (FEV1), forced vital capacity (FVC), FEV1/FVC, maximum midexpiratory flow, peak expiratory flow, and exercise capacity with a significant increase in the ratio of residual volume/total lung capacity [16].

CDH has health and economic influences on children and their families. The financial cost of intensive utilization of the health system by the surviving CDH children is continuously increasing with reduced productivity of families caring for these children and a marked reduction in quality of life (QOL) for CDH children and their families [17].

Recently, with a continued existence rate, long-standing evaluations have been considered in children with CDH, and this is where the QOL plays a key role in this issue [18]. The World Health Organization stated that QOL is a wide-range concept impacted in multiple dimensions by the individual's physical, psychological, social, and personal status and their association with relevant attributes of their surroundings [19]. A previous study concluded that the malformation severity of CDH may affect QOL negatively [20]. A recent study has found that exercise training improves respiratory functions and QOL in asthmatic children [21].

Different modalities of physical therapy have been evaluated in children with different respiratory disorders including aerobic exercise training [21], chest physical therapy [22], and inspiratory muscle training [23]. However, rarely have studies evaluated the impacts of respiratory muscle training on respiratory function, exercise capacity, functional performance, and QOL in school-aged children with postoperative CDH. Regarding that, our study was conducted to evaluate the impacts of the respiratory muscle training on respiratory functions, maximal exercise capacity, functional performance, and QOL in children with postoperative CDH hypothesizing that respiratory muscle training could have a useful impact on respiratory functions, exercise capacity, and QOL among those children.

## 2. Materials and Methods

### 2.1. Study Design

Between April 2019 and January 2020, this randomized controlled clinical trial was conducted. It was accomplished at the outpatient physiotherapy clinic at Prince Sattam bin Abdulaziz University. The ethical clearance was attained from the local institutional review board of the physiotherapy department (No. RHPT/018/055). All procedures were fulfilled in accordance with the ethical standards of the 1964 Declaration of Helsinki and its updates.

### 2.2. Subjects

Forty children with postoperative CDH were recruited from the pediatric surgical departments of the King Khalid Hospital and other referral hospitals in Al-Kharj, Saudi Arabia. Participants were included in the study if their ages ranged from 9 to 11 years, had BMI between 20 and 25 kg/m^2^, were diagnosed with CDH-associated respiratory distress within the first days of life (high-risk CDH), and have undergone an operation immediately after birth. Children who have a paraesophageal diaphragmatic defect, a diaphragmatic eventration, were unable to tolerably conduct the study procedures, or had any other serious anomalies were excluded.

### 2.3. Randomization and Blinding

**Figure 1:**
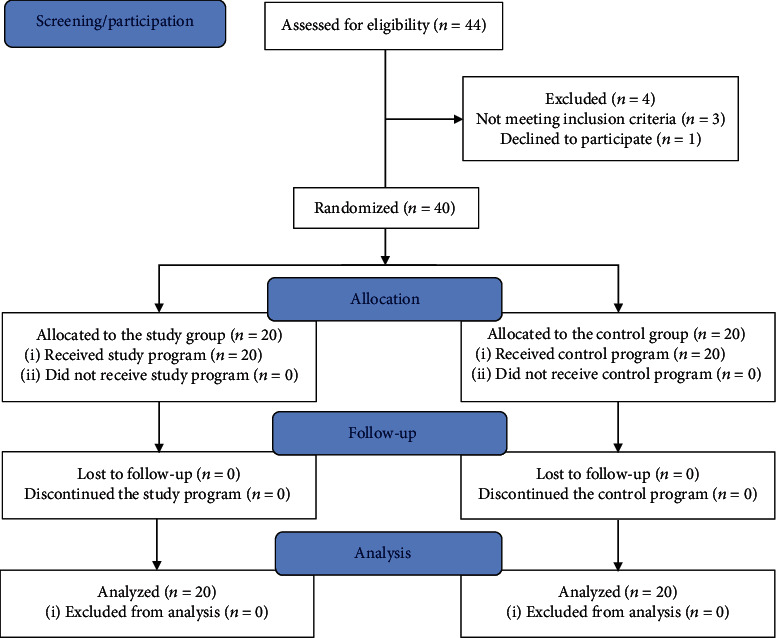
The flow diagram of the study participants through the trial.

From the forty-four children assessed for eligibility, three did not meet the inclusion criteria of the study and one of the parents had refused to enroll his child in the study without a specific reason. Utilizing SPSS version 22 (IBM Corp., Armonk, NY, USA), forty children were randomized before initiating the study procedures into the study group that conducted a program of inspiratory muscle training combined with incentive spirometer exercise, and the control group that conducted a program of incentive spirometer exercise alone. The parents or caregivers of the children were informed of the procedures of the study, and they were instructed to sign written consent forms before initiating the study procedure. The examiner was blinded to the group assignments.  Figure 1 demonstrates the CONSORT flowchart of the study.

### 2.4. Sample Size Estimation

Using VO_2_max as the primary outcome in the study, the sample size has been estimated. A prior study has evaluated the effect of aerobic exercise on exercise capacity in school-aged children and approved the significant difference of the mean VO_2_max of 5 mL/kg/min at least [21]. Based on this difference and the study objective to realize 80% power with type I error of 0.05, the present study required thirty-four children for the two study groups. Hence, the study included forty children to account for the 20% dropout.

### 2.5. Outcome Measures

Respiratory functions, maximal exercise capacity, functional performance, and quality of life were assessed before and immediately after treatment by an independent researcher who was not aware of the group treatment.

#### 2.5.1. Respiratory Functions

The respiratory functions were evaluated utilizing Spirolab III (SDI Diagnostics Inc., USA), the tests were conducted at a defined time between 09:00 am and 13:00 pm to limit diurnal variety. Using the spirometer, the youngsters were instructed to breathe 2-3 discretionary tidal breaths in and out, and to breathe profoundly (quick and profound) with lips fixed firmly around the mouthpiece. At that point, they were instructed to blow air through the mouthpiece as quickly as could be allowed and maintain blowing until no air is left to breathe out. Amid instructions, a few expressions could be utilized, for example, “sucking on a straw” for a profound motivation, “smothering birthday candles” for a powerful lapse, and “continue blowing and continue blowing” to finish exhaling until no air is left. The test was repeated three times to achieve reliable findings. The PC screen was displayed to the children frequently during the test to motivate the children to keep on performing the test [24–26]. The predicted values of forced vital capacity (FVC%) and forced expiratory volume in one second (FEV1%) were recorded before and after twelve weeks of the study program.

#### 2.5.2. Maximal Exercise Capacity

The maximal exercise capacity was evaluated through a cardiopulmonary exercise test (CPET) using the Bruce treadmill test. Parents or children's caregivers attended the test procedures. The Bruce test protocol comprised 3 min stages of increasing speed and intensity on a treadmill. Before initiating the test, the children were allowed to familiarize themselves with the mouthpiece and treadmill for 3 min. Each child was encouraged to continue until the point of severe fatigue. Heart rate and oxygen saturation were monitored using finger pulse oximetry. The maximal oxygen uptake (VO_2_max) and the ratio of minute ventilation (VE) to carbon dioxide production (VCO_2_) were recorded before and after twelve weeks of the study program [27, 28].

#### 2.5.3. Functional Performance

Functional performance was measured using the 6 min walk test (6-MWT). It is a valid and reliable modality to examine functional performance. Before conducting the 6-MWT, children and their parents were informed and educated about the purpose of the test and were shown the start and endpoints and instructed also to avoid hopping, running, or jumping during the test. Each child was instructed to walk through a 50-meter straight corridor over a period of six minutes, while the examiner closely followed them with a stopwatch. The distance in meters within the 6-MWT was recorded before and after twelve weeks of the study program [29].

#### 2.5.4. Quality of Life

Quality of life was assessed using a 23-item pediatric quality of life inventory (PedsQL). This instrument was validated to assess children and adolescents with acute or chronic diseases [30, 31]. The PedsQL questionnaire includes 4 domains (physical, social, emotional, and school functions). It provides child self-report for children aged ≥8 years with a 5-point Likert scale (0 means never, and 4 means almost always). Each response scores 0%, 25%, 50%, and 100% regarding ranges from never to always. The responses to the 23 items create a mean score for the 4 domains and a mean score for overall PedsQL [32].

### 2.6. Intervention

The study group was recruited to conduct a program of inspiratory muscle training combined with an incentive spirometer exercise. The control group was recruited to conduct the same incentive spirometer training.

#### 2.6.1. Incentive Spirometer Training

Incentive spirometer training was conducted using a flow-centered incentive spirometer (Triflow II, Respirogram, India). The device is made up of plastic material; it has three balls connected to a tube, and it also has a mouthpiece. Each child was informed to sit quietly in a relaxed position for some time and concentrate on his/her breathing. Consequently, each child was instructed to take a deep breath and to hold the flow-based incentive spirometer by one hand, while the other hand holds the mouthpiece and the tube. Children were asked to take 3-4 easy and slow breaths, then they were asked to inhale through the spirometer slowly and maximally to make the ball in the cylinder rise as high as possible, after that inspiration was held for at least 2-3 seconds before normally exhaling without the mouthpiece. These steps were repeated 5 times, and each child was informed to take a rest for about 60 seconds. This procedure was repeated for a total of 30 min thrice/week for 12 consecutive weeks [33].

#### 2.6.2. Inspiratory Muscle Training

Inspiratory muscle training was conducted using a Threshold IMT Breathing trainer (Respironics, Cedar Grove, NJ, USA). Inspiratory resistance was adjusted through a spring-loaded valve presented in the device. Each session lasted for 30 min through six inspiratory cycles. Each cycle lasted for 4 min of resisted respiration. The rest period was 60 s after each cycle, which is aimed at improving the strength of the respiratory muscles. During the last cycle, each child was encouraged to breathe as frequently as possible with the aim of improving muscle endurance. Throughout the exercise training, the threshold load was 40% of the maximal inspiratory pressure estimated during the child's assessment before starting the exercise session. The inspiratory muscle training was conducted thrice/week for 12 consecutive weeks [23].

### 2.7. Statistical Analysis

SPSS for Windows (V.22, IBM Corp., Armonk, NY, USA) was utilized for analyzing the collected data. Descriptive analysis was performed using means ± standard deviations (SD). The Shapiro-Wilk test was utilized for assessing the normal distribution of the data. The Student *t*-test was used in inferential statistics. The intergroup changes were analyzed utilizing the unpaired *t*-test, whereas the intragroup changes were analyzed utilizing the paired *t*-test. The level of significance was considered at *p* ≤ 0.05.

## 3. Results

Forty CDH children (24 boys and 16 girls) completed the study program. The study group comprised 20 children (11 boys and 9 girls), whereas the control group comprised 20 children (13 boys and 7 girls). As detailed in Table 1, demographic data (age, gender, height, weight, and BMI) showed no significant intergroup difference (*p* > 0.05). The mean values of the outcome measures including respiratory functions, maximal exercise capacity, function performance, and QOL showed no significant intergroup pretreatment differences (FVC%, *p* = 0.891; FEV1%, *p* = 0.887; VO_2_max, *p* = 0.871; VE/VCO_2_ slope, *p* = 0.752; 6-MWT, *p* = 0.203; and PedsQL, *p* = 0.509) as detailed in Table 2.

Regarding the posttreatment analysis intragroup, the study group showed significant improvements in the study outcome measures (FVC%, *p* < 0.001; FEV1%, *p* = 0.002; VO_2_max, *p* = 0.008; VE/VCO_2_ slope, *p* = 0.002; 6-MWT, *p* < 0.001; and PedsQL, *p* < 0.001), whereas the control group showed no significant changes (FVC%, *p* = 0.053; FEV1%, *p* = 0.107; VO_2_max, *p* = 0.290; VE/VCO_2_ slope, *p* = 0.291; 6-MWT, *p* = 0.292; and PedsQL, *p* = 0.632) as presented in Table 2.

**Figure 2:**
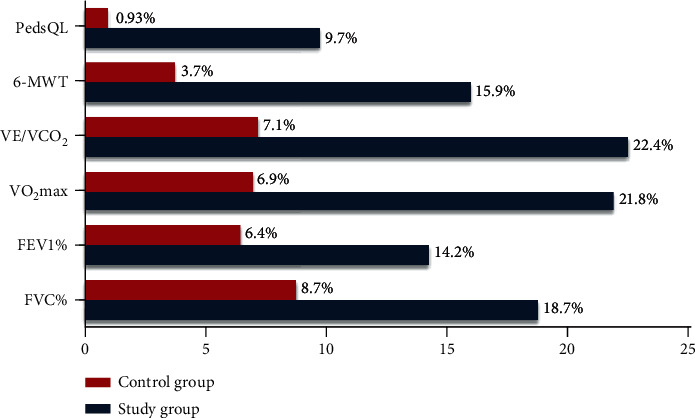
The percent of changes in the two groups posttreatment.

The percent of changes in the study group (FVC% = 18.7%; FEV1% = 14.2%; VO_2_max = 21.8%; VE/VCO_2_ slope = 22.4%; 6 − MWT = 15.9%; and PedsQL = 9.7%) versus the control group (FVC% = 8.7%; FEV1% = 6.4%; VO_2_max = 6.9%; VE/VCO_2_ slope = 7.1%; 6 − MWT = 3.7%; and PedsQL = 0.93%) are expressed in Figure 2. For intergroup analysis, significant differences were illustrated in all outcome measures posttreatment (FVC%, *p* = 0.019; FEV1%, *p* = 0.047; VO_2_max, *p* = 0.036; VE/VCO_2_ slope, *p* = 0.033; 6-MWT, *p* = 0.028; and PedsQL, *p* = 0.015) which favours the study group as detailed in Table 2.

## 4. Discussion

This randomized control study is aimed at evaluating the impacts of respiratory muscle training on respiratory functions, maximal exercise capacity, functional performance, and QOL in children with postoperative CDH, hypothesizing that respiratory muscle training could have a useful impact on respiratory functions, exercise capacity, and QOL among these children. The study findings verified our hypothesis and showed that inspiratory muscle training improved FVC%, FEV1%, VO_2_max, VE/VCO_2_, 6-MWT, and PedsQL among CDH children.

CDH makes abdominal organs move into the chest cavity leading to abnormal development of the lungs which may cause pulmonary hypoplasia and pulmonary hypertension, which are major determinants of morbidity and mortality in those children [2]. Respiratory training has been performed for those children in order to improve respiratory functions, exercise capacity, functional performance, and QOL. The results showed improvement in respiratory functions, exercise capacity, functional performance, and QOL in the study group compared with the control group.

The current study outcomes were supported by the results of Turchetta et al., who assessed whether physical activity could influence the performance and perception of dyspnea in CDH children operated for high-risk CDH. They found that CDH children who were more active preserved a higher level of performance with less perception of dyspnea and effort compared to children who were less active [34]. Also, Moawd et al. concluded that inspiratory muscle training improves inspiratory muscle strength and aerobic capacity in patients experiencing obstructive sleep apnea [35].

To our knowledge, no previous studies have evaluated the effect of respiratory muscle training on respiratory functions, maximal exercise capacity, functional performance, and QOL in CDH children although some authors have evaluated the effect of respiratory exercises on similar cases. Lima et al. have studied the effect of inspiratory muscle training on respiratory distress in asthmatic children and found that specific inspiratory muscle training plus breathing exercises were effective adjuvant therapies in the treatment of respiratory problems in asthmatic children as both maximum inspiratory and expiratory pressure have been increased significantly, with a corresponding reduction in airway obstruction, and exercise capacity has also been increased [23]. As stated by Toussaint-Duyster et al., CDH children have a marked reduction in exercise capacity at school age [36]. Thus, inspiratory muscle training may be effective in improving exercise capacity in children with CDH.

The strength and endurance of the respiratory muscles including the diaphragm and accessory muscles may be improved following inspiratory muscle training [37, 38]. Being functionally and morphologically skeletal muscles, the respiratory muscles can react to exercise in a similar way when appropriate physiological resistance is applied [39, 40]. Improving the strength of the respiratory muscles delays the development of diaphragmatic fatigue, enhances ventilator competence, decreases the required blood flow throughout the exercise, and consequently improves functional capacity [41, 42].

In spite of the positive outcomes of the current study, it may be limited in terms of generalizability and the external validity of the results are limited by the small sample size. Selection bias could affect the results as the participants do not broadly reflect all children with congenital diaphragmatic hernia. The measures of pulmonary function are largely dependent on the level of the child's effort and motivation. Future studies are needed to examine the mechanism of how respiratory muscle training affects physiological indicators. It is essential to note that the results of this study are limited to school-aged CDH children from 9 to 11 years old and should be applied carefully to other age groups. Also, our results cannot confirm the long-term effect of respiratory muscle training. Therefore, upcoming studies should consider a long-term follow-up (6-12 months) after the end of treatment program.

## 5. Conclusions

Respiratory muscle training may improve respiratory functions, maximal exercise capacities, functional performance, and QOL in children with postoperative CDH. Clinical commendations have to be considered to include respiratory muscle training in pulmonary rehabilitation programs in the children with a history of CDH.

## Figures and Tables

**Table 1:** Demographic data of the study children.

Variables	Study group (*n* = 20)	Control group (*n* = 20)	*p* value
Age (years)	10.1 ± 0.7	9.8 ± 0.8	0.215
Gender, boys/girls (*n*)	11/9	13/7	0.518
Height (cm)	137.5 ± 5.9	135.8 ± 5.4	0.347
Weight (kg)	42.6 ± 4.2	41.8 ± 4.7	0.573
BMI (kg/m^2^)	23.2 ± 3.8	23.5 ± 3.6	0.799
Duration of hospitalization (days)	52.4 ± 7.5	49.8 ± 7.2	0.271
Medications, *n* (%)			
Low/moderate dose of inhaled corticosteroid	5 (25)	8 (40)	0.311
Long-acting *β*2-agonist + low/moderate dose of inhaled corticosteroid	15 (75)	12 (60)	

Significant level at *p* < 0.05. BMI: body mass index.

**Table 2:** Intra- and intergroup changes of outcome variables pre- and posttreatment.

Variables	Study group (*n* = 20)	Control group (*n* = 20)	*p* value
FVC (% pred.)			
Pre-	78.5 ± 9.8	78.9 ± 10.5	0.891
Post-	93.2 ± 7.4	85.8 ± 11.3	0.019
*p* value	<0.001	0.053	
FEV1 (% pred.)			
Pre-	72.3 ± 8.5	72.7 ± 9.2	0.887
Post-	82.6 ± 7.2	77.4 ± 8.8	0.047
*p* value	0.002	0.107	
VO_2_max (mL/kg/min)			
Pre-	40.3 ± 7.4	40.7 ± 8.1	0.871
Post-	49.1 ± 7.9	43.5 ± 8.4	0.036
*p* value	0.008	0.290	
VE/VCO_2_ slope			
Pre-	31.6 ± 7.1	30.9 ± 6.8	0.752
Post-	24.5 ± 5.8	28.7 ± 6.2	0.033
*p* value	0.002	0.291	
6-MWT			
Pre-	421.3 ± 38.7	438.4 ± 44.5	0.203
Post-	488.5 ± 42.8	454.6 ± 51.2	0.028
*p* value	<0.001	0.292	
PedsQL (overall score)			
Pre-	75.41 ± 12.5	72.84 ± 11.9	0.509
Post-	82.73 ± 11.2	73.52 ± 11.7	0.015
*p* value	<0.001	0.632	

FVC: forced vital capacity; FEV1: forced expiratory volume in one second; VO_2_max: maximal oxygen uptake; VE/VCO_2_: minute ventilation/carbon dioxide production; 6-MWT: six-minute walk test; PedsQL: pediatric quality of life inventory.

## Data Availability

The data involved in this study is available from the corresponding author upon request and privacy-related data of the patients will not be provided.
